# Supplementary material for: A Comprehensive Molecular and Clinical Analysis of the piRNA Pathway Genes in Ovarian Cancer
Source: Cancers (Basel). 2020 Dec 22;13(1):4. doi: 10.3390/cancers13010004 (PMC7792616; doi:10.3390/cancers13010004)
Supplement: Supplementary file 1 [file cancers-13-00004-s001.zip › cancers-983649 revised supp.docx]

**Supplementary Materials:**

A Comprehensive Molecular and Clinical Analysis of the piRNA Pathway Genes in Ovarian Cancer

Eunice Lee, Noor A. Lokman, Martin K. Oehler, Carmela Ricciardelli and Frank Grutzner

**Table 1.** Clinicopathological characteristics of benign and high grade serous ovarian cancer cohort used in the Fluidigm qRT-PCR.

| **Benign serous cystadenomas (n=17)** | | |
| --- | --- | --- |
| **Age** | **Median (range)** | 60 (25-75) |
| **High grade serous ovarian carcinomas (n=29)** | | |
| Age at Diagnosis | Median (range) | 59 (38-84) |
| Histological Grade | High | 29 |
| FIGO stage | Stage I  Stage II  Stage III | 4  8  17 |

**Table 2.** Clinicopathological characteristics of chemosenstitive and chemoresistant ovarian cancer patient cohort used in the Fluidigm qRT-PCR.

| **Chemoresponse** | **Patient** | **Age at Diagnosis** | **Diagnosis** | **Stage** | **Grade** |
| --- | --- | --- | --- | --- | --- |
| Sensitive | 1 | 66 | Serous carcinoma of ovary/ peritoneum | 3c | 3 |
|  | 2 | 72 | Serous papillary carcinoma of the ovary | 3c | 3 |
|  | 3 | 58 | Serous carcinoma of the ovary | 3c | 3 |
|  | 4 | 85 | Serous papillary carcinoma of the peritoneum | 3c | 3 |
|  | 5 | 58 | Serous papillary carcinoma of the peritoneum | 1c | 3 |
| Resistant | 6 | 85 | Recurrent serous carcinoma of the ovary | 3c | 3 |
|  | 7 | 47 | Recurrent serous carcinoma of the ovary | 3c | 3 |
|  | 8 | 47 | Recurrent serous papillary carcinoma of the peritoneum | 3c | 3 |
|  | 9 | 81 | Recurrent serous papillary carcinoma of the peritoneum | 4 | 3 |
|  | 10 | 69 | Recurrent serous papillary carcinoma of the ovary | 3a | 3 |
|  | 11 | 46 | Recurrent serous papillary carcinoma of the ovary | 3c | 3 |
|  | 12 | N/A | Recurrent serous carcinoma of the ovary | 1c | 3 |

**Table 3.** Taqman assays used in qRT-PCR.

| **Gene** | **Assay ID** |
| --- | --- |
| *PIWIL1* | Hs01041737_m1 |
| *PIWIL2* | Hs01032720_m1 |
| *PIWIL3* | Hs00908825_m1 |
| *PIWIL4* | Hs00381509_m1 |
| *DDX4* | Hs00987125_m1 |
| *HENMT1* | Hs00989130_m1 |
| *MAEL* | Hs00262601_m1 |
| *PLD6* | Hs00381651_m1 |
| *TDRD1* | Hs00229805_m1 |
| *TDRD9* | Hs00403678_m1 |
| *GUSB* | Hs00939627_m1 |
| *TBP* | Hs00427620_m1 |

**Table S4.** Affymetrix ID of piRNA pathway genes assessed in the Kaplan-Meier plotter.

| **Gene** | **Affymetrix ID** |
| --- | --- |
| *PIWIL1* | 214868_at |
| *PIWIL2* | 220686_s_at |
| *PIWIL4* | 230480_at |
| *DDX4* | 221630_s_at |
| *HENMT1* | 225841_at |
| *MAEL* | 229475_at |
| *PLD6* | 227037_at |
| *TDRD1* | 221018_s_at |
| *TDRD9* | 228285_at |

**Table S5.** Primers used in RT-PCR.

| **Gene** | **Sense** | **Anti-sense** |
| --- | --- | --- |
| $\beta$*-actin* | 5’-GACGACATGGAGAAAATCTG-3’ | 5’-ATGATCTGGGTCATCTTCTC-3’ |
| *PIWIL2* | 5’-TATGAGGTGAACCACTGTCTAC-3’ | 5’-GTTGAGAACACAGACATAATGC-3’ |
