# Supplementary material for: A Comprehensive Molecular and Clinical Analysis of the piRNA Pathway Genes in Ovarian Cancer
Source: Cancers (Basel). 2020 Dec 22;13(1):4. doi: 10.3390/cancers13010004 (PMC7792616; doi:10.3390/cancers13010004)
Supplement: Supplementary file 1 [file cancers-13-00004-s001.zip › cancers-983649.WB.pdf]

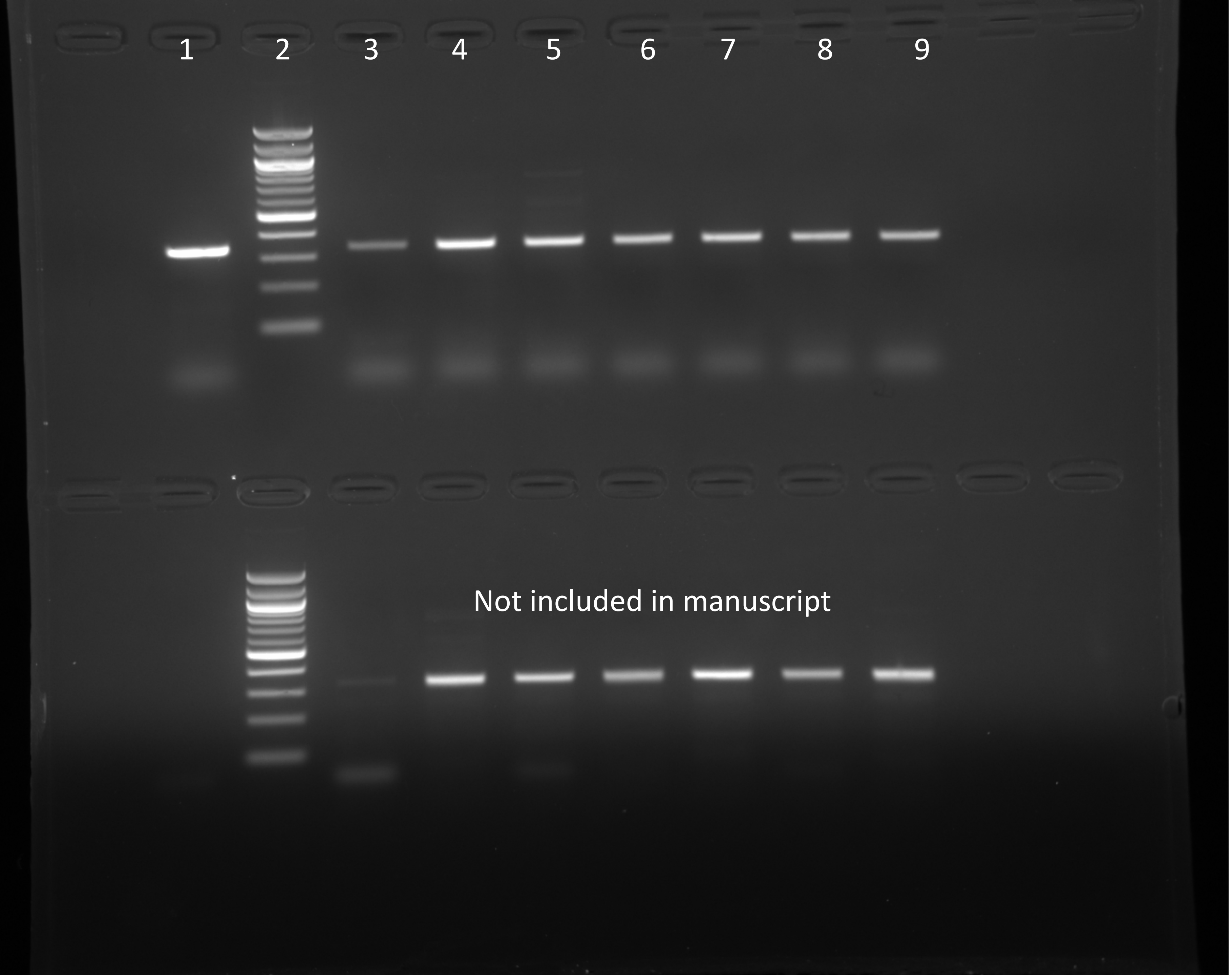

Figure 4. *PIWIL2* expression
Lanes:
1: Positive control (not included in manuscript image)
2: 100bp ladder
3: Untreated
4: 50 FSH
5: 100 FSH
6: 25 LH
7: 50 LH
8: 25 LH + 50 FSH
9: 50 LH + 100 FSH

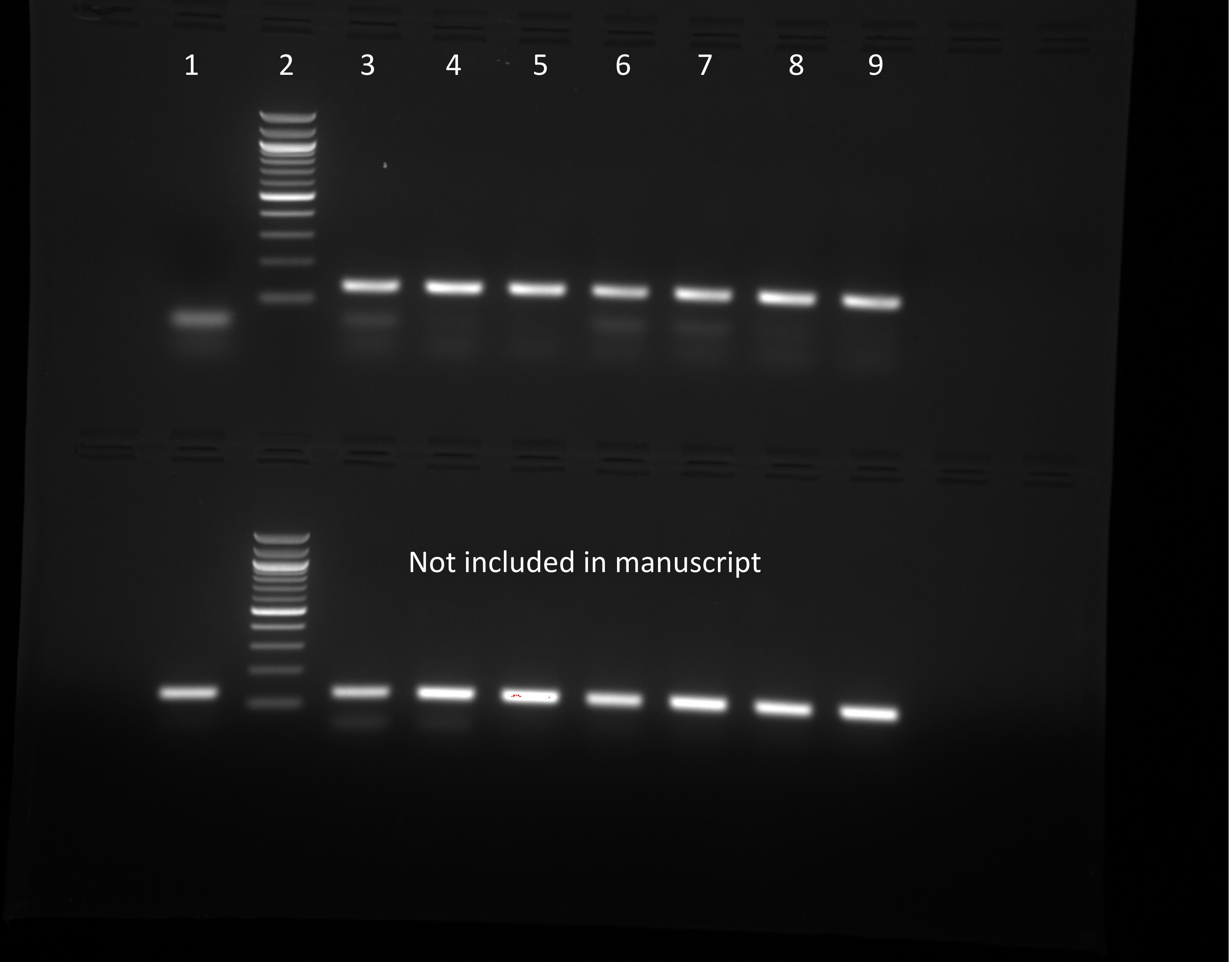

Figure 4. *β-actin* expression
Lanes:
1: Negative control (not included in manuscript image)
2: 100bp ladder
3: Untreated
4: 50 FSH
5: 100 FSH
6: 25 LH
7: 50 LH
8: 25 LH + 50 FSH
9: 50 LH + 100 FSH
